# Effect of cutting process parameters on fatigue properties of quenched and tempered 42CrMo steel

**DOI:** 10.1038/s41598-026-38185-4

**Published:** 2026-02-02

**Authors:** Ke Tang, Jiang Zhu, Binhao Yin, Qing Hao, Bicheng Guo, Jiyao Li, Shiqi Chen, Jiashun Gao, Zhilong Xu, Fasheng Zhong

**Affiliations:** 1 Sichuan Huadian Gongxian Power Generation Co., Ltd., Yibin, 644500 China; 2 College of Marine Equipment and Mechanical Engineering, Jimei University, Xiamen, 361000 China; 3 School of Marine Mechatronics, Xiamen Ocean Vocational College, Xiamen, 361000 China; 4 Chengyi College, Jimei University, Xiamen, 361000 China; 5 Engineering Research Center of Anti-Fatigue Manufacturing for Marine Equipment, Xiamen, 361000 China; 6 Xiamen Xgma Crec Heavy Machinery Co.,Ltd., Xiamen, China

**Keywords:** 42CrMo steel, Cutting operations, Surface roughness, Surface residual compressive stress, Metal fatigue life, Engineering, Materials science

## Abstract

Quenched and tempered 42CrMo steel is a commonly used material for critical components such as high-strength bolts, spindles, and transmission shafts, where fatigue failure induced by alternating loads is its primary failure mode. Machining, as a pivotal step connecting upstream and downstream processes in the manufacturing chain, significantly influences the fatigue performance of metal parts through the surface integrity it generates. Under wet cutting conditions, this study systematically conducted experiments to investigate the effect of cutting parameters—cutting speed (*v*), feed rate (*f*), and depth of cut (${\mathrm{a}}_{\mathrm{p}}$)—on fatigue performance, The influence of cutting parameters on surface roughness and residual stress was analyzed. Based on this analysis, a weighted standardization method integrating both roughness and residual stress was proposed for the comprehensive evaluation of fatigue life. The feasibility of this method was subsequently verified and analyzed through experiments. The results indicate that cutting speed exerts the most significant influence on surface roughness, while the distribution of residual stress is also considerably affected by cutting speed. Rotating bending fatigue tests and fracture analysis demonstrate that crack initiation and propagation result from the synergistic effect of surface roughness and residual stress, with surface residual compressive stress and its gradient distribution playing a dominant role in determining fatigue life. The novel weighted criterion proposed in this study exhibits strong consistency with fatigue life, providing both experimental evidence and a theoretical tool for optimizing cutting parameters and enhancing the service performance of 42CrMo critical components.

## Introduction

 Machinery manufacturing forms the bedrock of national industrial development, wherein the fatigue performance of critical components in high-end equipment—such as shafts, gears, and connecting rods—directly impacts the overall reliability and service life of the machinery. These components frequently operate under complex alternating loads, with failure often stemming from the initiation and propagation of fatigue cracks at or below the surface. 42CrMo steel is a typical medium-carbon quenched and tempered alloy structural steel. Owing to its high strength, good toughness and excellent fatigue properties, it is widely used in the manufacture of critical mechanical components such as shafts, gears and connecting rods. During actual service, these components frequently endure alternating loads, and their fatigue life directly impacts the overall reliability and service life of the machinery. Therefore, enhancing the fatigue properties of 42CrMo steel components holds significant engineering importance. Common methods for enhancing the fatigue properties of 42CrMo steel currently include heat treatment phase transformation strengthening^1^, laser shock peening^2^, laser shot peening^3^, and machining^4^. However, the fatigue performance of components depends not only on the material’s inherent mechanical properties but is also significantly influenced by surface integrity parameters^5^ formed during processing, such as surface roughness^6^, residual stresses^7^, and microstructure^8^. As one of the primary machining methods for final shaping, the selection of cutting process parameters directly determines the physical state of the workpiece surface^9–11^. Particularly for high-strength materials such as quenched and tempered 42CrMo steel, the combined effects of mechanical and thermal influences during machining significantly impact the plastic deformation of surface layers, residual stress distribution, and microstructural evolution. This, in turn, influences the initiation and propagation behaviour of fatigue cracks^12,13^.

At present, a large number of studies have focused on the influence of cutting parameters on surface roughness and residual stress^14–16^. However, there is still insufficient systematic research on the intrinsic relationship between surface integrity parameters and fatigue performance under the synergistic effect of multiple parameters, especially for the quenched and tempered 42CrMo material under wet cutting conditions.

Therefore, this study systematically analyzed the influence of cutting speed, feed and depth of cut on the surface roughness and residual stress by a single factor cutting experiment, screened out multiple groups of typical process parameters, and further carried out the rotational bending fatigue test to explore the influence mechanism of surface integrity parameters on the fatigue life, so as to provide experimental basis and theoretical support for optimizing the cutting process of 42CrMo parts and improving their fatigue performance.

## Materials and methods

### Materials

This study investigated a quenched and tempered 42CrMo steel. The heat treatment process for the 42CrMo steel consisted of austenitizing at 845 ℃, followed by oil quenching and tempering in air at 540 ℃. Its chemical composition is provided in Table 1, and the basic mechanical properties are listed in Table 2.

**Table 1:** Chemical composition of 42CrMo steel(wt%).

Ingredients	C	Si	Mn	*P*	S	Ni	Cr	Mo	Cu	V	Fe
content	0.41	0.24	0.74	0.012	0.023	0.03	1.15	0.21	0.08	0.01	Other

**Table 2:** Basic mechanical properties of 42CrMo steel.

Yield strength/MPa	Tensile strength/MPa	Section shrinkage rate	Elongation rate	Hardness/HV
1080	900	0.45	0.12	260∼300

### Methods

**Fig. 1:**
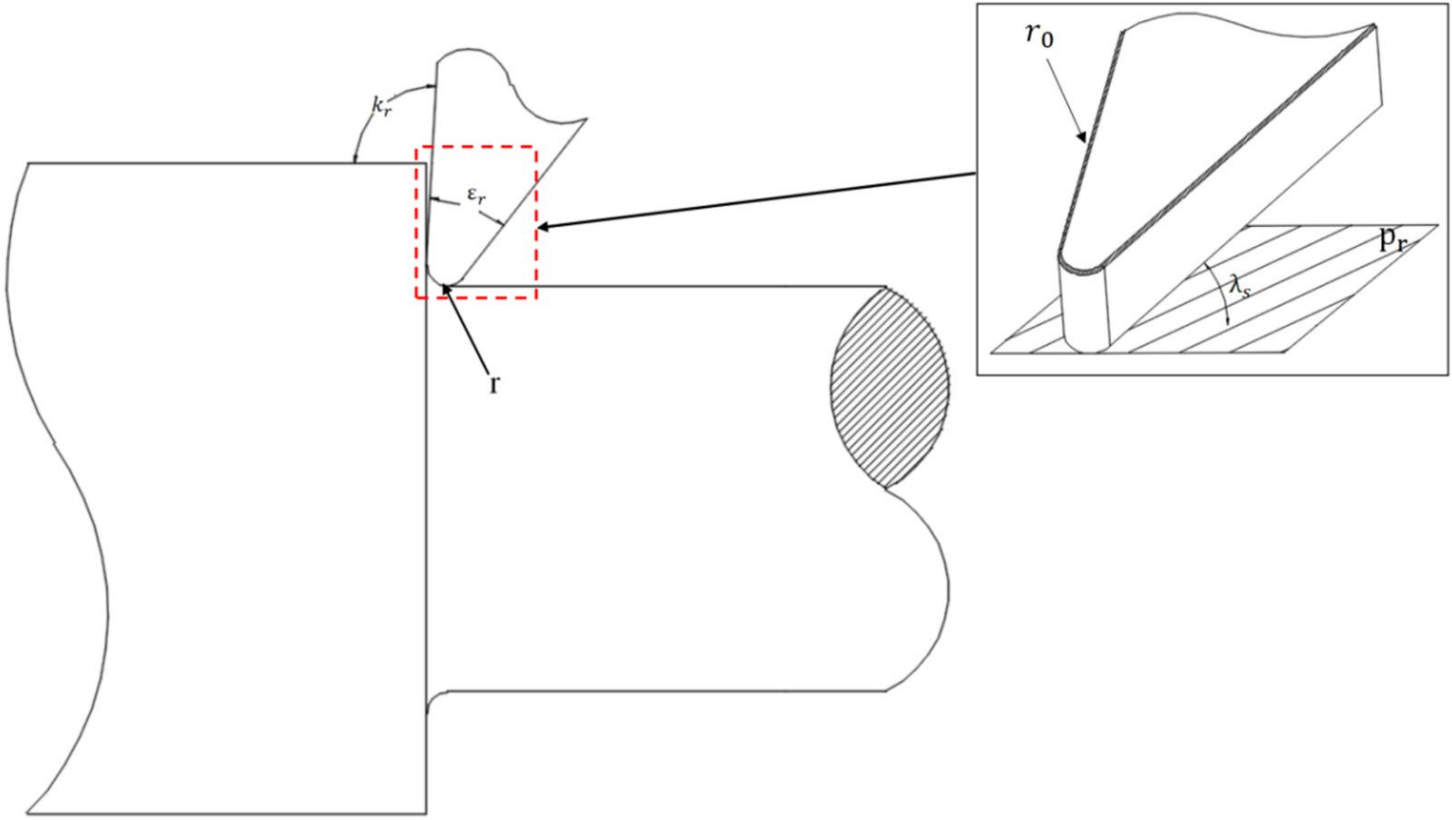
Main parameters of the cutting tool.

This study first employed a single-factor controlled experiment to machine Φ20 × 100 mm specimens. The lathe is the WEINO CNC lathe TC-T200, with a VNMG160404-GF cutting tool. The principal tool parameters are shown in Fig. 1.

The primary parameters of the cutting tool are as follows: tool tip angle (*εr*) = 35 °, tool nose radius (*r*) = 0.4 mm, principal cutting edge angle (*κr*) = 93 °, reference plane (*pr*), tool cutting edge inclination angle (*λ*_*s*_) = 6 °, and cutting edge rounding radius (*r₀*) = 0.014 mm.

**Fig. 2:**
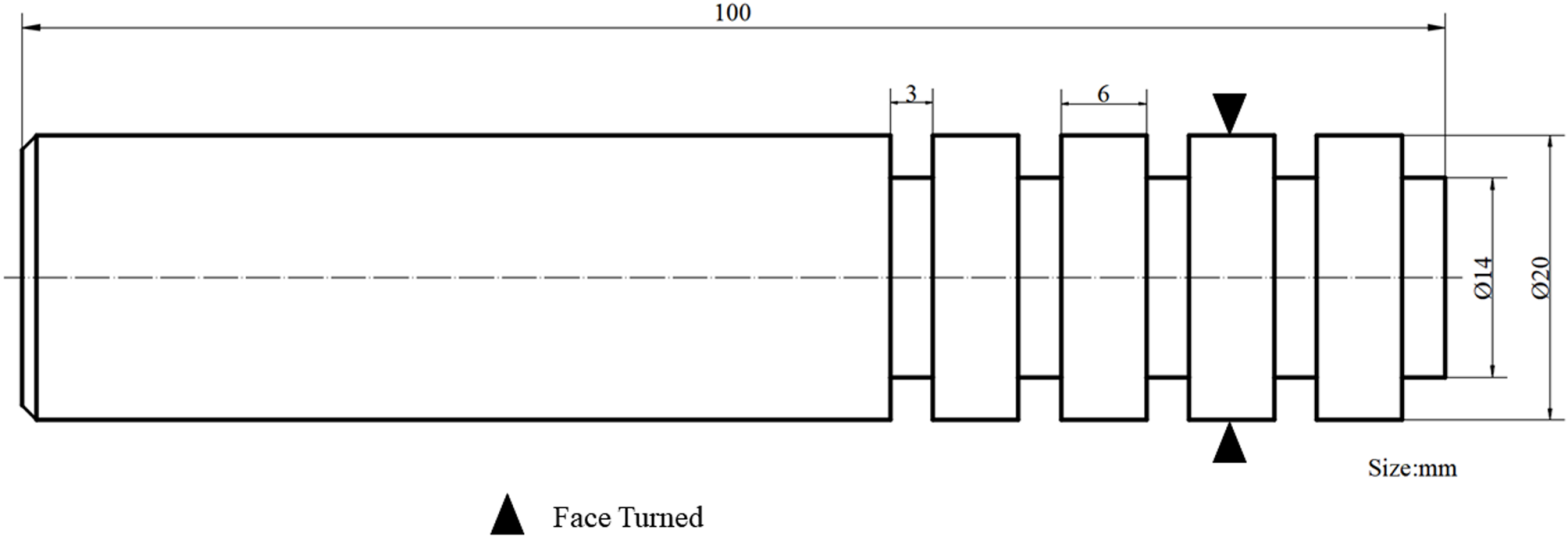
Single-factor test sample size.

The processing size of the sample is shown in Fig. 2. Four rings are processed on a sample, and each ring corresponds to a processing surface with different parameters. Surface roughness and residual stress measurements were conducted on the turned surfaces using a Time3200 roughness measuring instrument and an HDS-I X-ray stress diffractometer. For each parameter set, three measurements were taken, and the average values along with the standard deviations of surface roughness and residual compressive stress are presented in Table 3.

**Table 3:** Single-factor experimental results.

Serial number	Cutting speed v (m/min)	Feed rate f (mm/rev)	Depth of cut $\:{\mathrm{a}}_{\mathrm{p}\text{}}$(mm)	Roughness value(µm)	Residual compressive stress(MPa)
T1	120	0.01	0.4	0.130 ± 0.011	693.3 ± 54.9
T2	120	0.02	0.4	0.139 ± 0.007	558.7 ± 27.3
T3	120	0.03	0.4	0.197 ± 0.016	341.3 ± 19.1
T4	120	0.04	0.4	0.255 ± 0.026	86.7 ± 20.6
T5	140	0.02	0.4	0.111 ± 0.005	646.3 ± 6.1
T6	120	0.02	0.4	0.208 ± 0.023	604.0 ± 19.5
T7	100	0.02	0.4	0.332 ± 0.128	599.0 ± 42.5
T8	80	0.02	0.4	0.391 ± 0.004	553.7 ± 28.6
T9	120	0.02	0.2	0.206 ± 0.007	649.0 ± 39.0
T10	120	0.02	0.4	0.272 ± 0.003	539.7 ± 11.0
T11	120	0.02	0.6	0.289 ± 0.016	543.0 ± 4.0
T12	120	0.02	0.8	0.307 ± 0.009	584.0 ± 21.4

## Results

### Roughness degree

**Fig. 3:**
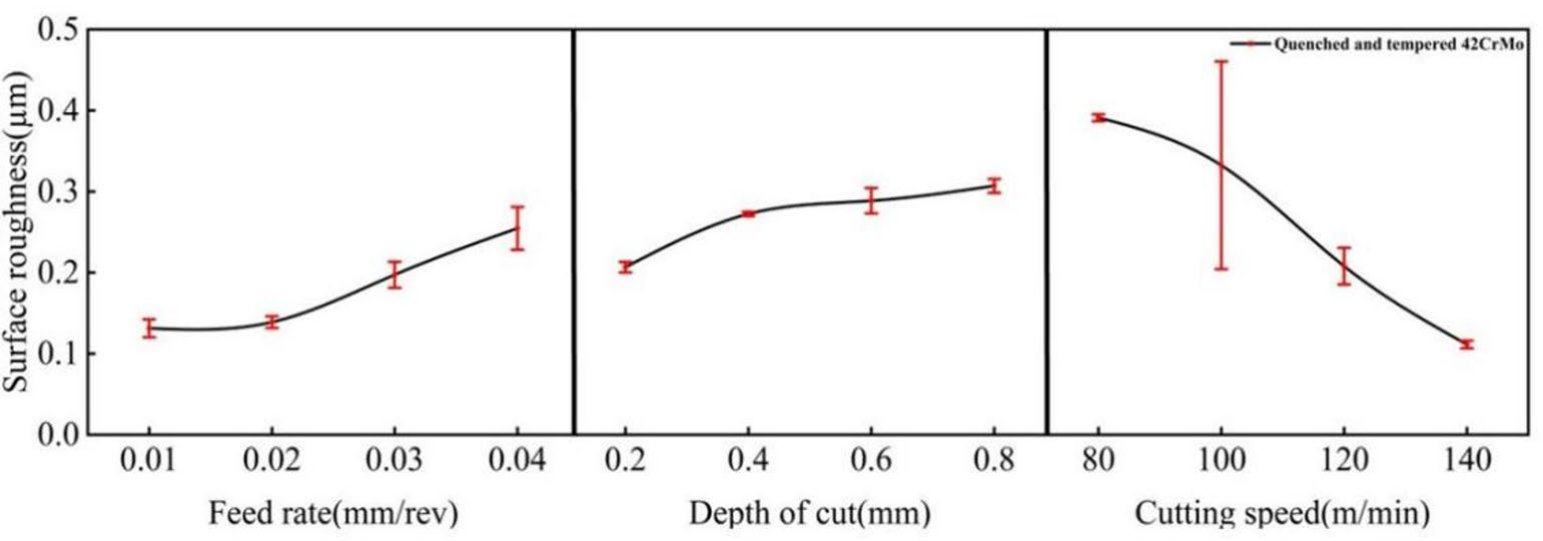
Relationship between feed, depth of cut, cutting speed and roughness value.

The article uses the arithmetic mean deviation Ra as the main parameter for evaluating surface roughness. It can be seen from Fig. 3 that the feed *f* has an important influence on the surface roughness. Assuming that the tool tip is absolutely sharp, the turned surface forms a ripple called residual height^17^.

The theoretical formula for the residual height is given by Eq. 1:

1 $$\:H=\frac{{f}^{2}}{8R}$$

Where H is the residual height, $\:{f}^{\:}$is the feed rate, and *R* is the tool nose radius.

According to the theoretical formula, the residual height (*H*) is positively correlated with the feed rate (*f*). Figure 3 confirms this overall trend, showing that surface roughness generally increases with *f.* A notable deviation, however, is the comparable roughness and fluctuation observed at *f* = 0.01 mm/rev and *f* = 0.02 mm/rev. This deviation is attributed to the detrimental effect of an excessively low feed rate: at *f* = 0.01 mm/rev, the cutting process produces powdery chips, which impairs surface formation and prevents the further improvement in roughness.

Furthermore, the cutting speed (*v*) also exerts a significant influence on the surface roughness, as shown in Fig. 3. Particularly within the range of 80 to 120 m/min, increasing the cutting speed markedly improves the surface roughness. This improvement occurs because, within the low to medium speed range, an increase in cutting speed typically leads to a slight reduction in cutting force. This reduction helps minimize elastic deformation and vibration in the machining system, thereby promoting stable cutting and resulting in a more uniform surface. However, when the cutting speed reaches 140 m/min, machine tool vibration may intensify and high temperatures can be generated, accelerating tool wear and potentially leading to a rougher surface. Nevertheless, in this study, the application of a cutting fluid environment substantially reduced the heat generated during high-speed machining. The combined action of high-speed cutting and the cutting fluid suppressed or eliminated the formation of a built-up edge, significantly improving surface quality and ultimately yielding a smoother surface finish.

The depth of cut has minimal impact on the roughness value and exerts no direct influence on the theoretical residual height. However, as shown in Fig. 3, increasing the depth of cut generally tends to slightly worsen the surface roughness. A greater depth of cut leads to a significant rise in cutting force, exacerbating the vibration and elastic deformation within the machine tool, tool, and workpiece system. This results in an unstable actual cutting process, uneven surface waviness on the machined part, and an increase in roughness values. However, when comparing the depth of cut of 0.4 mm, 0.6 mm, and 0.8 mm, although all three show a gradual increase, the upward trend is slow, and the overall fluctuation remains insignificant.

### Residual stress

**Fig. 4:**
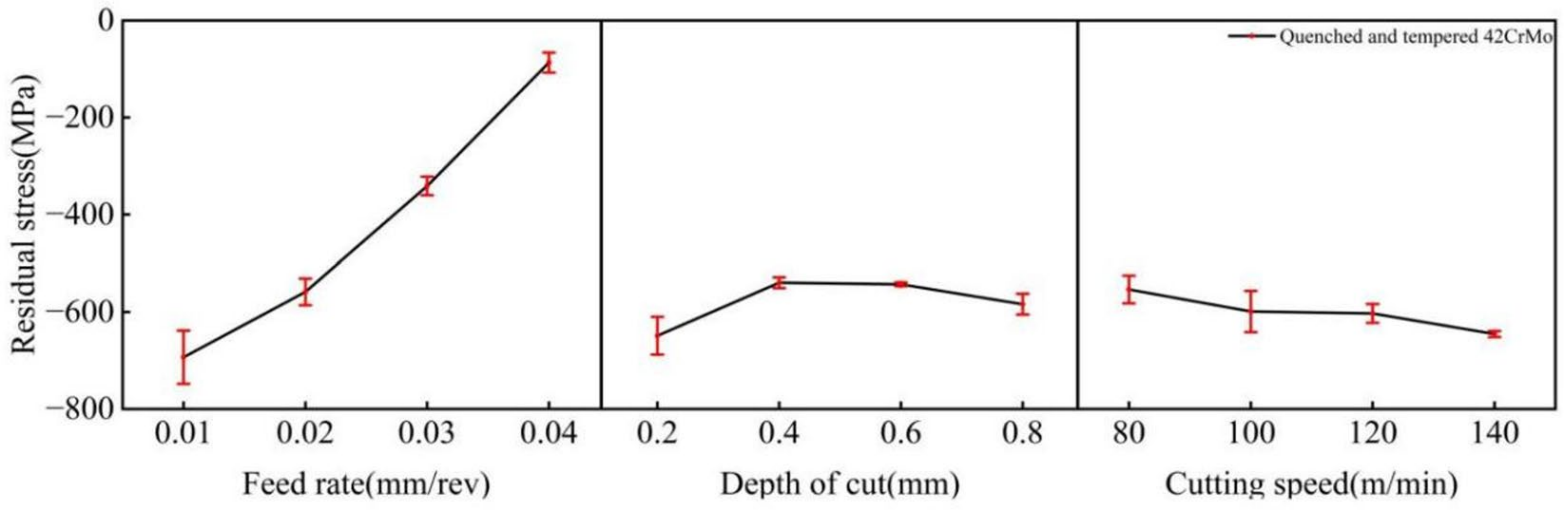
Relation between feed, depth of cut, cutting speed and residual stress.

An increase in the feed rate enhances the extrusion effect of the tool on the workpiece, resulting in a deeper plastic deformation layer and a significant rise in cutting force. However, as shown in Fig. 4, the residual compressive stress in this experiment exhibited a decreasing trend with increasing feed rate. This phenomenon can be attributed to the notable reduction in the effective contact zone between the tool and the workpiece surface at higher feed rates, leading to non-uniform extrusion and plastic deformation.

The impact of the depth of cut on residual stress is not as pronounced and direct as that of cutting speed and feed rate. Increasing the depth of cut typically deepens the plastic deformation layer, thereby tending to generate compressive stresses, but its influence is less significant than that of the feed rate. As illustrated in Fig. 4, in this experiment, as the depth of cut increased, the residual compressive stress initially decreased and then increased. This could be attributed to the fact that when the depth of cut was 0.2 mm, the chips formed well, heat dissipation was excellent, and a substantial amount of heat was carried away by the chips, with the mechanical effect dominating and resulting in significant compressive stress. At a depth of cut of 0.4 mm, the chips wrapped around the workpiece, and the generated heat was not effectively removed by the chips. Although the cutting fluid carried away some heat, a certain thermal effect still occurred, leading to a partial release of the residual compressive stress on the surface and an upward trend. When the depth of cut ranged from 0.6 to 0.8 mm, chip breaking was good, the mechanical effect again dominated, the temperature rise of the workpiece was controllable, and the residual compressive stress continued to increase.

The cutting speed (*v*) exhibits a notable influence on the residual stress. Within the tested range of 80 to 140 m/min, compressive residual stresses were consistently generated. The magnitude of this residual compressive stress increased with the cutting speed, rising from − 553.7 MPa to −617.7 MPa. This trend is attributed to the dominance of mechanical plastic deformation at lower speeds, where the extrusion and friction from the tool induce compressive plastic deformation in the surface layer, resulting in compressive residual stresses. At higher speeds, although the cutting temperature increases, the substantial heat dissipation by the cutting fluid suppresses the thermal effect. Consequently, the mechanical plastic deformation remains predominant over the thermal effect, leading to a further increase in the residual compressive stress.

### Weighted standard value

Measure the surface roughness and residual surface stress of the experimental test sample. Then, apply min-max normalization to map the measured values of surface roughness and residual surface stress to the interval [0, 1], ensuring that all indicators are in the same order of magnitude. Since fatigue life decreases with an increase in roughness value and increases with an increase in residual compressive stress, the roughness value is inversely normalized, while the residual compressive stress is positively normalized.

Equation 2 presents the calculation for the positive indicator normalization:

2 $$\:{\mathrm{X}}_{\mathrm{p}}=\frac{{X}_{1}\mathrm{-}{\mathrm{X}}_{\mathrm{1}\mathrm{min}}}{{\mathrm{X}}_{\mathrm{1}\mathrm{max}}\mathrm{-}{\mathrm{X}}_{\mathrm{1}\mathrm{min}}}$$

Where $\:{\mathrm{X}}_{\mathrm{p}}$ is the normalized value, $\:{\mathrm{X}}_{1}$ is the original roughness value, and $\:{\mathrm{X}}_{\mathrm{1}\mathrm{min}}$ and $\:{\mathrm{X}}_{\mathrm{1}\mathrm{max}}$ are the minimum and maximum values, respectively, within the entire data set for the roughness indicator.

Equation 3 defines the normalization calculation for the negative indicator:

3 $$\:{\mathrm{X}}_{\mathrm{r}}=\frac{{X}_{2max}-{X}_{2}}{{\mathrm{X}}_{\mathrm{2}\mathrm{max}}\mathrm{-}{\mathrm{X}}_{\mathrm{2}\mathrm{min}}}$$

Where $\:{\mathrm{X}}_{\mathrm{r}}\:$is the normalized value, $\:{\mathrm{X}}_{2}$ is the original residual compressive stress value, and $\:{\mathrm{X}}_{\mathrm{2}\mathrm{max}}$ and $\:{\mathrm{X}}_{\mathrm{2}\mathrm{min}}$ are the maximum and minimum values, respectively, within the data set for this indicator.

Taking into account the influence weights of roughness and residual compressive stress on fatigue life^18–20^, a weight ratio of 35% for roughness and 65% for residual compressive stress is assigned.

Equation 4 represents the calculation formula for the weighted standard value.

4 $$X_{i} = 0.35*X_{p} + 0.65*X_{r}$$

Where $\:{\mathrm{X}}_{\mathrm{i}}\:$is the weighted standardized value, $\:{\mathrm{X}}_{\mathrm{p}}$ is the normalized value of the positive indicator, and $\:{\mathrm{X}}_{\mathrm{r}}$ is the normalized value of the negative indicator.

**Table 4:** Weighted standardization results of cutting parameters.

Serial number	Cutting speed v (m/min)	Feed rate f (mm/rev)	Depth of cut ${\mathrm{a}}_{\mathrm{p}}$(mm)	Reverse indicator	Positive indicator	Weighted standard value
T1	120	0.01	0.4	0.932	1.000	0.973
T2	120	0.02	0.4	0.900	0.778	0.821
T3	120	0.03	0.4	0.693	0.420	0.516
T4	120	0.04	0.4	0.486	0.000	0.170
T5	140	0.02	0.4	1.000	0.923	0.949
T6	120	0.02	0.4	0.654	0.853	0.783
T7	100	0.02	0.4	0.211	0.845	0.623
T8	80	0.02	0.4	0.000	0.770	0.501
T9	120	0.02	0.2	0.661	0.927	0.834
T10	120	0.02	0.4	0.425	0.745	0.633
T11	120	0.02	0.6	0.364	0.752	0.616
T12	120	0.02	0.8	0.300	0.820	0.638

**Fig. 5:**
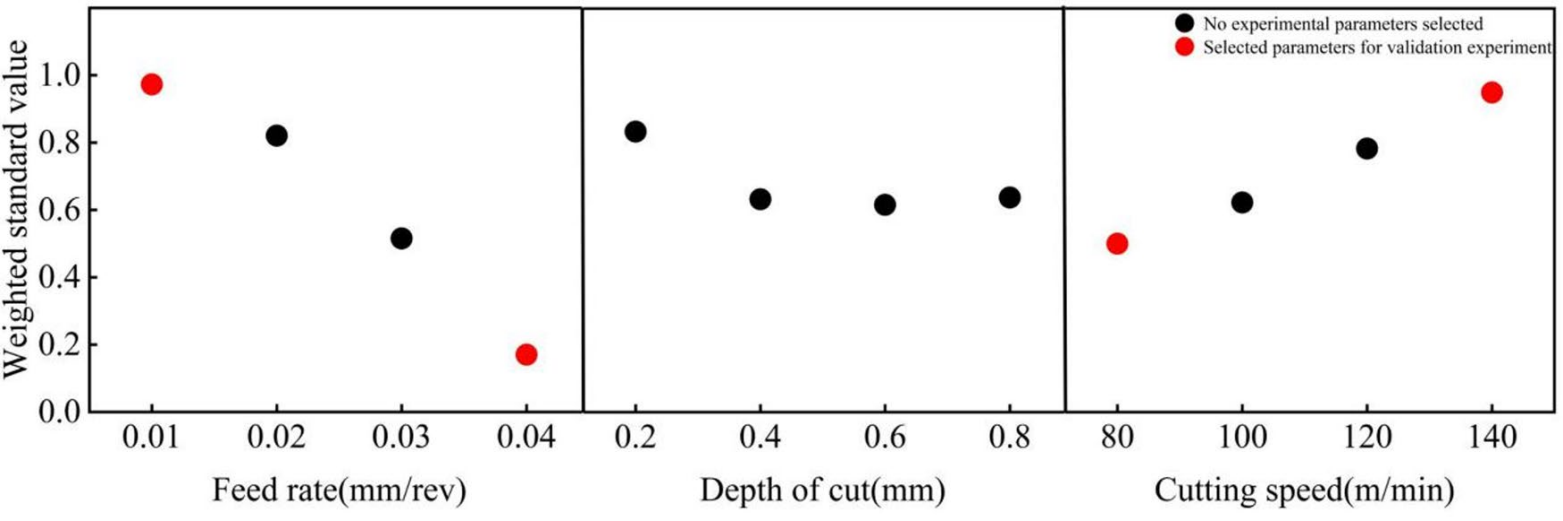
Weighted standardization experiment screening.

As shown in Fig. 5, by observing the relationship between the weighted standardization value and the cutting speed *v*, feed rate *f*, as well as the depth of cut, it is found that the feed rate and cutting speed have a significant impact on the weighted standardization value. As shown in Figs. 4 and 5, an increase in feed rate leads to greater roughness and reduced residual compressive stress, thereby significantly reducing the weighted standardization value.

With the increase in cutting speed, while the residual compressive stress does indeed rise, the overall fluctuation remains insignificant. However, the roughness exhibits a noticeable downward trend, consequently leading to an upward trend in the weighted standard value.

Depth of cut has little influence on the weighted standard value (except when the depth of cut is 0.2 mm, where both stress and roughness exhibit sharp fluctuations). This is because changes in depth of cut have a negligible effect on roughness and residual compressive stress.

To validate the accuracy of the weighted standard value in characterizing fatigue life, four parameter sets—T1, T4, T5, and T8 (indicated by red circles in Fig. 5)—were selected based on the criterion that their weighted standard values were closest to the maximum or minimum. Rotating bending fatigue tests were subsequently conducted on these sets for comparative analysis.

## Verification experiment

**Fig. 6:**
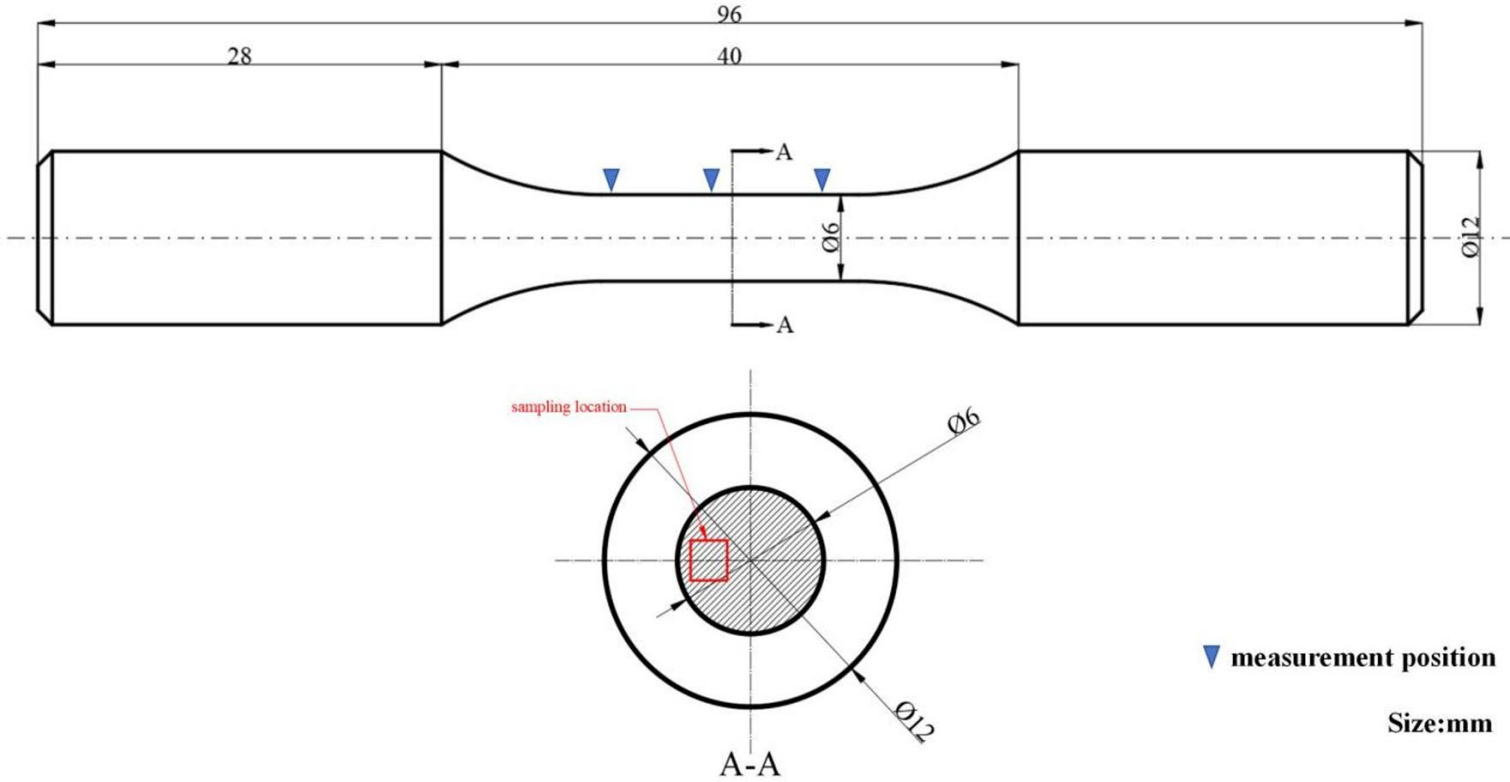
Processing dimensions of fatigue specimens and sampling positions for microstructure analysis.

To validate the feasibility of using the weighted standard value for predicting fatigue life, the four parameter sets (T1, T4, T5, and T8) selected from Table 4 were employed for verification experiments. The geometry of the rotating bending fatigue test specimen is illustrated in Fig. 6. For each parameter set, six specimens were manufactured. The first specimen from each set was used for measuring surface roughness, residual stress, and stress gradient. The second specimen was dedicated to microstructural analysis. The remaining three specimens were subjected to rotating bending fatigue tests.

For roughness and stress characterization, measurements were taken at three different locations on the working section of the specimen. The microstructural sample was extracted from a subsurface region near the working section surface via wire electrical discharge machining, producing a specimen of approximately 2 × 2 mm. The extracted sample was then ground, polished, and etched with a 2% nitric acid solution. Microstructural observation was conducted using a Keyence LSM900 laser scanning confocal microscope. A QBWP-6000 J rotating bending fatigue testing machine was employed for the fatigue tests. A stress amplitude of 680 MPa was applied at a loading frequency of 50 Hz. The relatively low frequency was selected to maintain stable specimen temperature during testing and to avoid interference from thermal effects. All tests were conducted under ambient temperature (~ 25 °C) in air without active temperature control. We prepared and tested three parallel fatigue specimens, and the results in the article are their average lifetimes. This comprehensive experimental procedure was designed to systematically validate the correlation between the proposed weighted standard value and the actual fatigue life.

### Roughness analysis

**Fig. 7:**
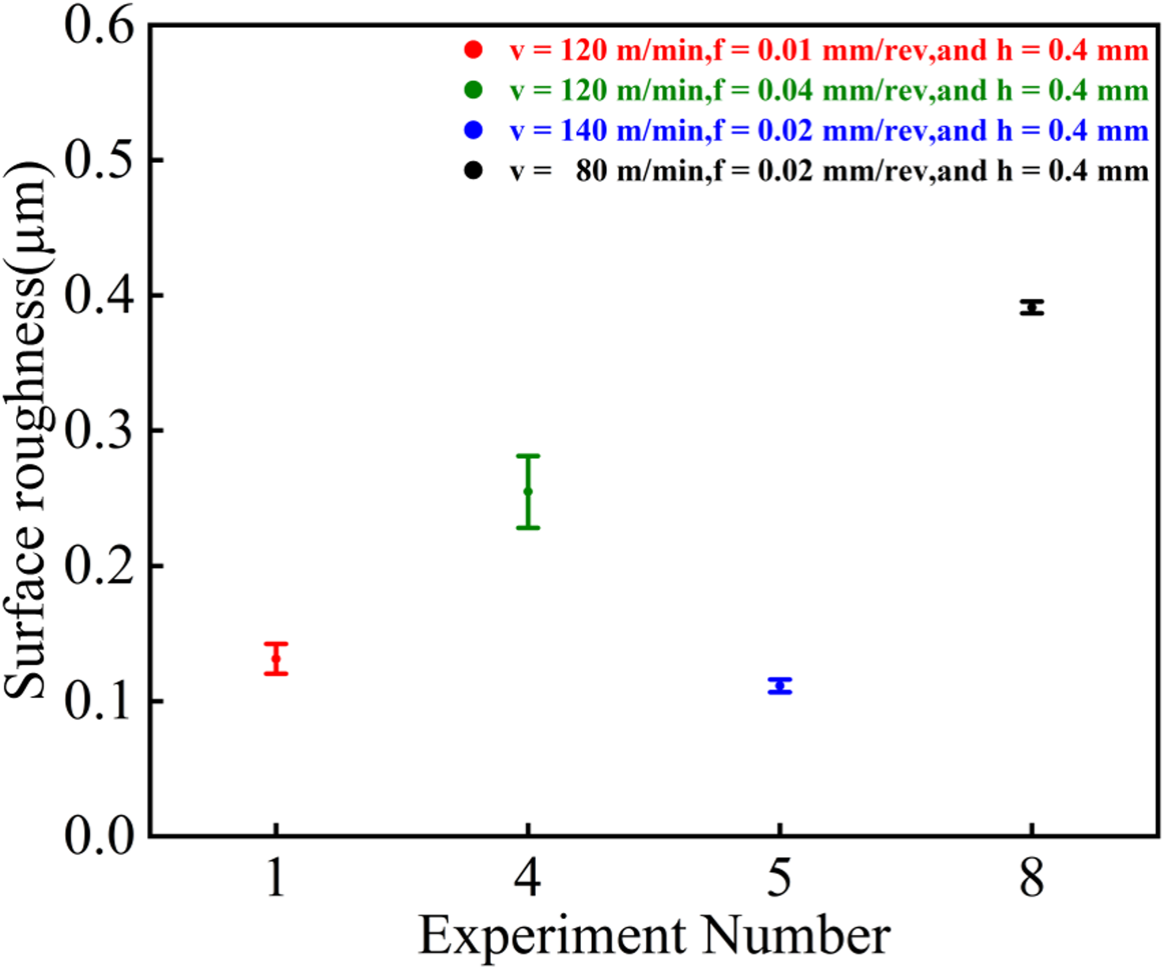
Surface roughness under different parameters.

As shown in Fig. 7, the T5 parameter set exhibited the best surface quality with an average roughness of 0.119 μm, while the T8 set showed the poorest surface quality with an average roughness of 0.372 μm. The T4 and T1 sets demonstrated average roughness values of 0.241 μm and 0.133 μm, respectively. Overall, the experimental results for these four parameter sets are in general agreement with the trends observed in the single-factor experiments.

### Analysis of residual compressive stress

**Fig. 8:**
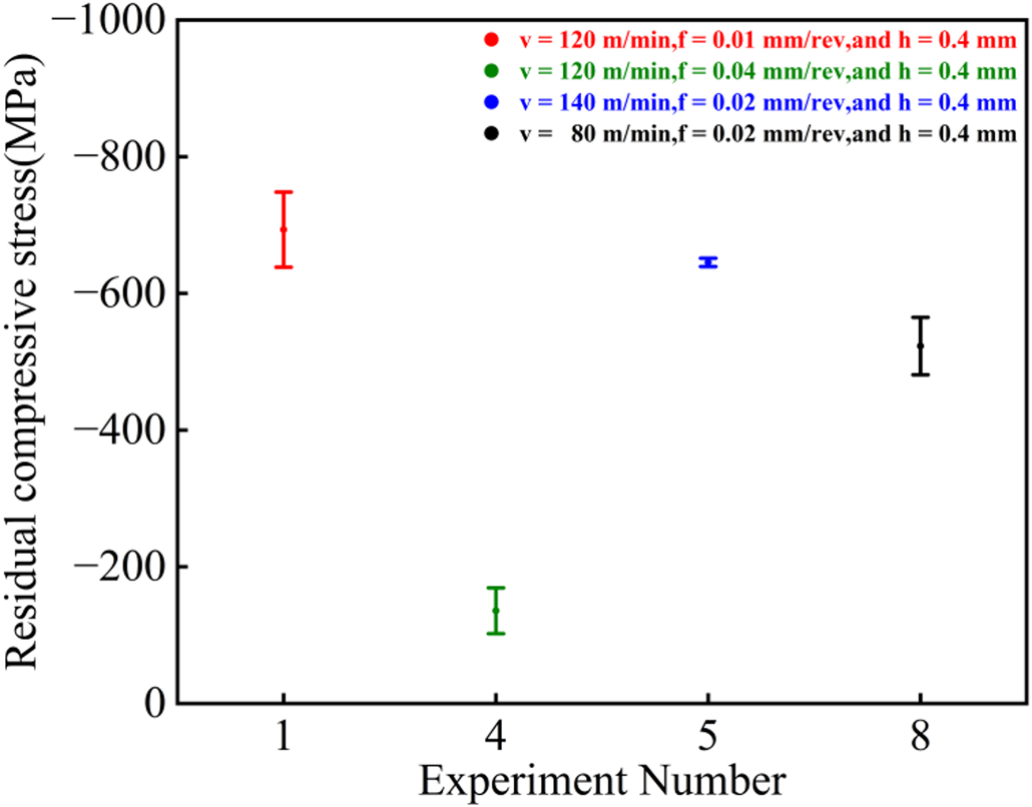
Surface residual compressive stress under different parameters.

As shown in Fig. 8, the T1 parameter set exhibited the highest surface residual compressive stress with an average value of −675.7 MPa, while the T4 set showed the lowest average value of −122.3 MPa. The average surface residual compressive stresses for the T5 and T8 sets were − 506.7 MPa and − 540.8 MPa, respectively. Although some variations were observed in the experimental data across the four parameter sets—likely attributable to machine tool vibrations induced by the relatively small diameter of the workpiece section—the overall results remain consistent with the trends identified in the single-factor experiments.

**Fig. 9:**
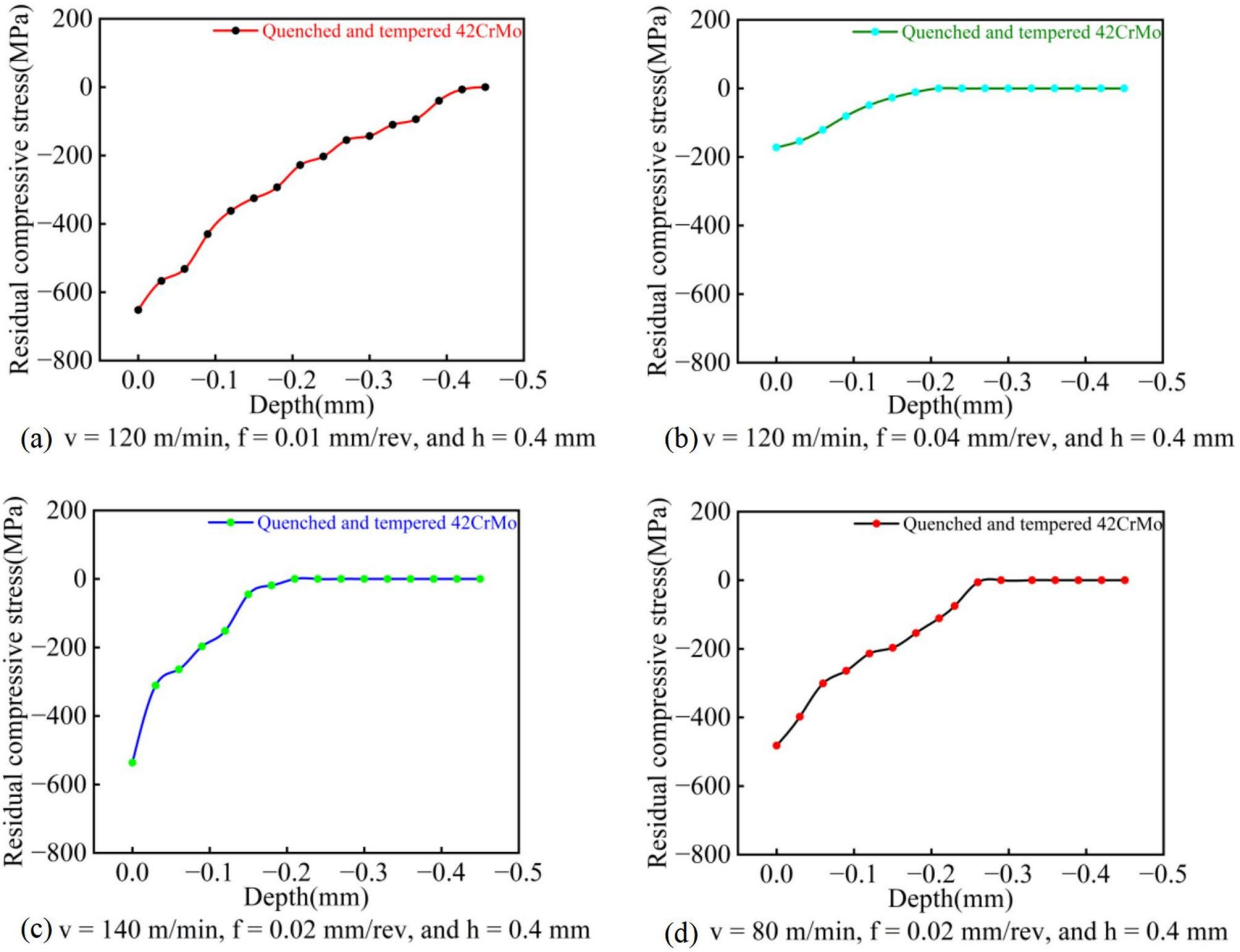
Residual compressive stress gradients under different parameters: (**a**–**d**) correspond to the T1, T4, T5, and T8 parameter sets, respectively.

As shown in Fig. 9a–d, the T1 parameter set produced a particularly favorable residual stress gradient. The maximum residual compressive stress reached − 652.7 MPa at the surface and gradually decreased with depth, transitioning to tensile stress at approximately − 450 μm. Compared to conventional turning processes^21^, this parameter combination generates a deeper layer of residual compressive stress. This enhanced gradient results from the synergistic effect of high cutting speed and low feed rate, which promotes more intensive mechanical-dominated plastic deformation. Simultaneously, the effective heat dissipation by the cutting fluid suppresses significant thermal effects that would otherwise relieve compressive stresses. Consequently, the predominance of mechanical effects over thermal effects yields an ideal stress profile characterized by high surface compressive stress and a deep compressive stress zone.

In contrast, although specimens machined with the T5 and T8 parameters exhibited considerable surface residual compressive stresses of −536 MPa and − 432 MPa, respectively, the depths of their compressive stress layers were relatively shallow, measuring only 0.18 mm and 0.26 mm. Furthermore, the residual stress gradient for the T4 parameter set showed more pronounced fluctuations, likely induced by machine tool vibration during processing. The T4 parameter set itself, characterized by a high feed rate, resulted in a comparatively low surface residual compressive stress of only − 172 MPa, which was insufficient to form a favorable stress gradient.

### Microstructure analysis

**Fig. 10:**
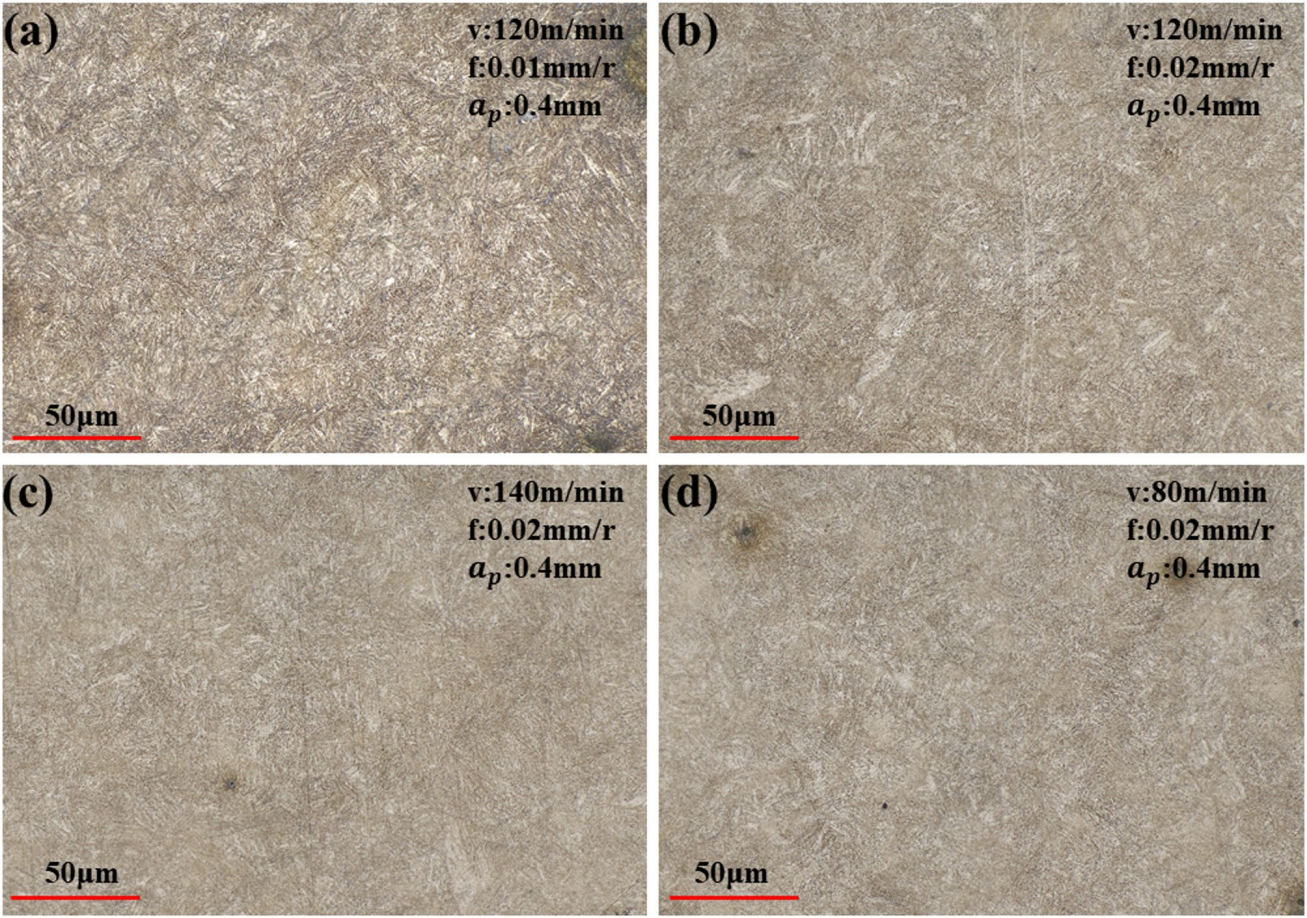
Microstructure under different parameters: (**a−d**) correspond to the T1, T4, T5, and T8 parameter sets, respectively.

As shown in Fig. 10a–d, all four parameter sets resulted in varying degrees of grain fragmentation, with the microstructure consistently exhibiting tempered lath martensite. However, comparative analysis reveals that the T1 parameter set induced the most substantial microstructural alterations, characterized by more severe grain fragmentation and notably enhanced surface grain refinement. In contrast, the T4, T5, and T8 parameter sets produced considerably less pronounced effects in terms of both grain refinement and microstructural density, with these three parameter sets demonstrating nearly identical microstructural characteristics.

### Analysis of bending fatigue life

**Fig. 11:**
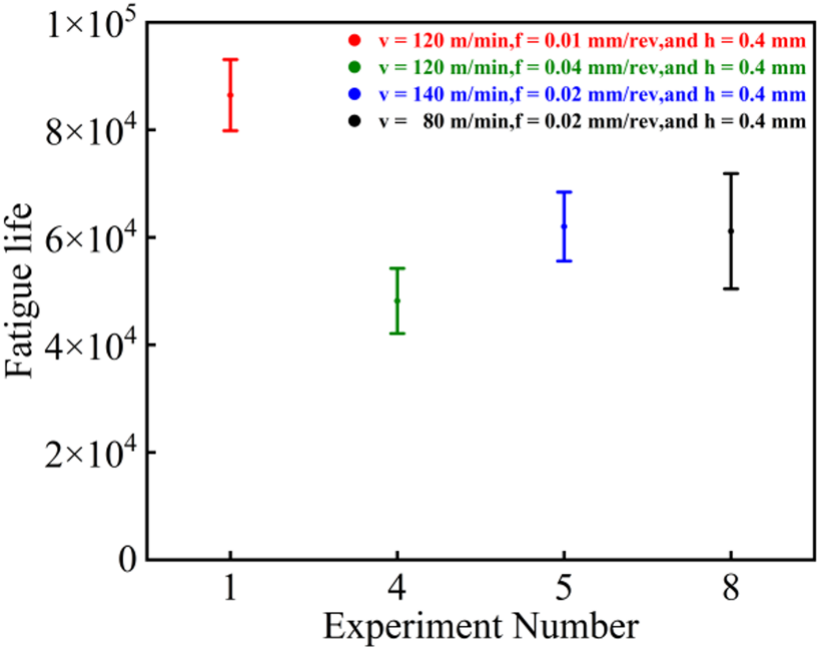
Bending fatigue life under different parameters.

As shown in Fig. 11, the T1 parameter set demonstrated the longest fatigue life in three repeated tests, achieving a maximum fatigue life of 95,220 cycles. This superior performance can be attributed to the combined effect of favorable surface characteristics. Firstly, as indicated in Fig. 7, the surface roughness under T1 parameters was the second best among the four sets, being only slightly higher than that of T4. Furthermore, as revealed in Figs. 8 and 9a, this parameter combination generated exceptionally high surface residual compressive stress along with a deeper compressive stress gradient. Since fatigue cracks typically initiate and propagate in regions of tensile stress^22,23^, the presence of surface residual compressive stresses and a deeper compressive stress gradient serve as the most beneficial factors for enhancing bending fatigue life^24^. These compressive stresses can effectively counteract the tensile stresses induced by external alternating loads, thereby suppressing or delaying both the initiation and propagation of micro-cracks. Even if micro-cracks have initiated, the compressive stress promotes crack tip closure, significantly reducing the crack propagation rate. As shown in Fig. 10a, the surface grain refinement induced by the T1 parameters resulted in work hardening of the surface material, enhancing both its strength and hardness. This moderate strain hardening is beneficial for impeding crack initiation^25^.

A comparison between the T5 and T8 parameter sets reveals that while the magnitudes of their surface residual compressive stresses are similar, T5 yields superior surface roughness whereas T8 produces a deeper compressive stress gradient. However, the rotating bending fatigue tests indicated only a marginal difference in the fatigue life between these two sets. This result further confirms that favorable surface residual compressive stress and its gradient distribution play a more dominant role in determining fatigue life than surface roughness.

**Fig. 12:**
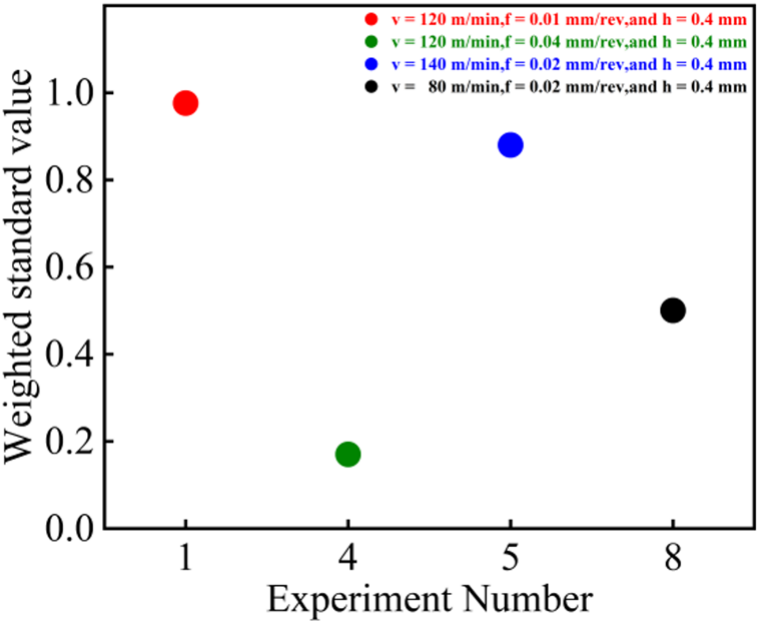
Weighted standard values under different parameters.

As illustrated in Figs. 12 and 11, the T1 parameter set, which possesses the highest weighted standard value (0.973), also demonstrates the optimal fatigue performance, achieving a maximum fatigue life of 95,220 cycles in three tests. Conversely, the T4 set, with the lowest weighted standard value (0.170), exhibits the shortest fatigue life. The weighted standard values for the T5 and T8 sets are 0.919 and 0.501, respectively, and their corresponding fatigue lives fall between those of T1 and T4. Notably, the fatigue life of T5 is slightly longer than that of T8, which aligns consistently with the trend indicated by their weighted standard values. This correspondence demonstrates that the weighted standard value effectively integrates the influence of surface roughness and residual compressive stress on fatigue life. The higher weighting assigned to residual stress (65%) compared to surface roughness (35%) underscores the dominant role of residual stress and its gradient distribution. The T1 parameter set, characterized by a high-magnitude residual compressive stress (−675.7 MPa) and a relatively deep stress gradient layer, significantly suppressed crack initiation and propagation. Although its surface roughness (0.130 μm) was not the lowest among the sets, it still achieved the longest fatigue life. In contrast, the T4 set, despite its relatively favorable roughness (0.255 μm), exhibited the poorest fatigue life due to its insufficient residual compressive stress (−122.3 MPa). The comparison between T5 and T8 further validates this observation: while T5 produced a superior surface roughness (0.111 μm), T8 generated a deeper compressive stress gradient. The comparable fatigue lives of these two sets indicate that the beneficial compensation provided by residual stress outweighs the improvement from surface roughness refinement. Therefore, the weighted standard value can be effectively used as a comprehensive evaluation indicator to reliably represent fatigue life.

### Fracture analysis

To thoroughly investigate the fatigue failure mechanisms of specimens under different cutting parameters, macro- and micro-scale analyses were performed on the fatigue fracture surfaces of four parameter sets: T1, T4, T5, and T8.

**Fig. 13:**
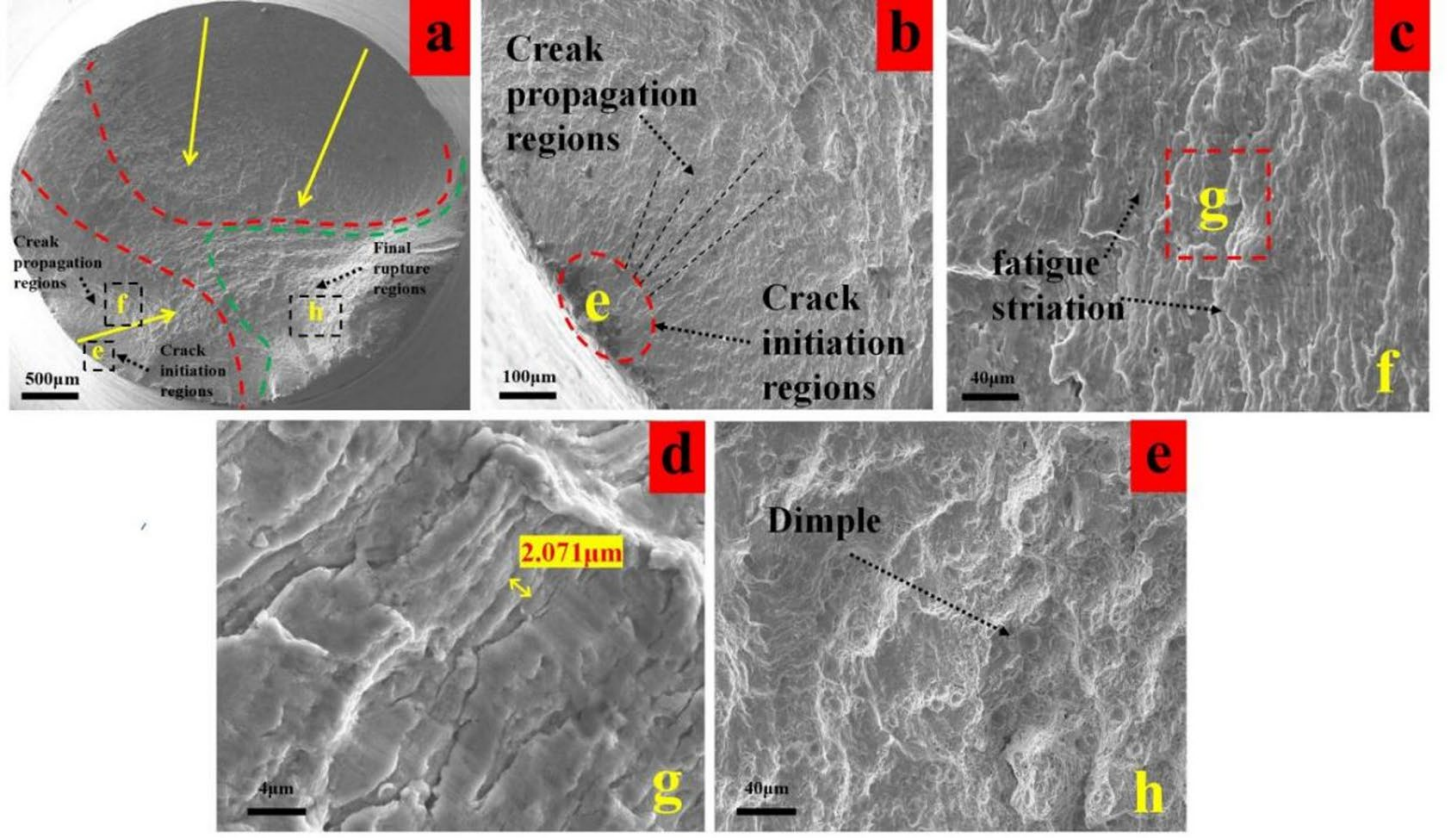
Fatigue fracture analysis of group T1 (**a**) macro-morphology of fracture, (**b**) enlarged view of crack initiation region, (**c**) fatigue striation of crack propagation zone, (f) dimples in the final rupture region.

As shown in Fig. 13a, the fracture surface of the T1 specimen macroscopically exhibits a distinct crack propagation zone, primarily formed by inward extension from three fatigue initiation zones. The fatigue area is uniformly distributed, with clearly visible crack propagation marks characterized by fine and continuous spacing (Fig. 13b). Although the fracture surface indicates a multi-crack initiation trend, the extended crack propagation path and prolonged duration are evident. This is attributed to the synergistic effect of high surface compressive residual stress and a deep residual stress gradient, which collectively suppressed crack propagation.

At the microscopic level, the propagation zone reveals fine and uniformly distributed fatigue striations (Fig. 13c,d). The spacing of these striations is positively correlated with the crack growth rate. The presence of closely spaced striations indicates that compressive residual stresses continuously hindered the advancement of the crack tip, significantly reducing the propagation rate. This serves as the key microscopic reason for the extended fatigue life of the T1 specimen.

The final fracture area is small and exhibits uniformly distributed fine dimples (Fig. 13e), suggesting that the material retained considerable plasticity even during the final ductile fracture stage.

**Fig. 14:**
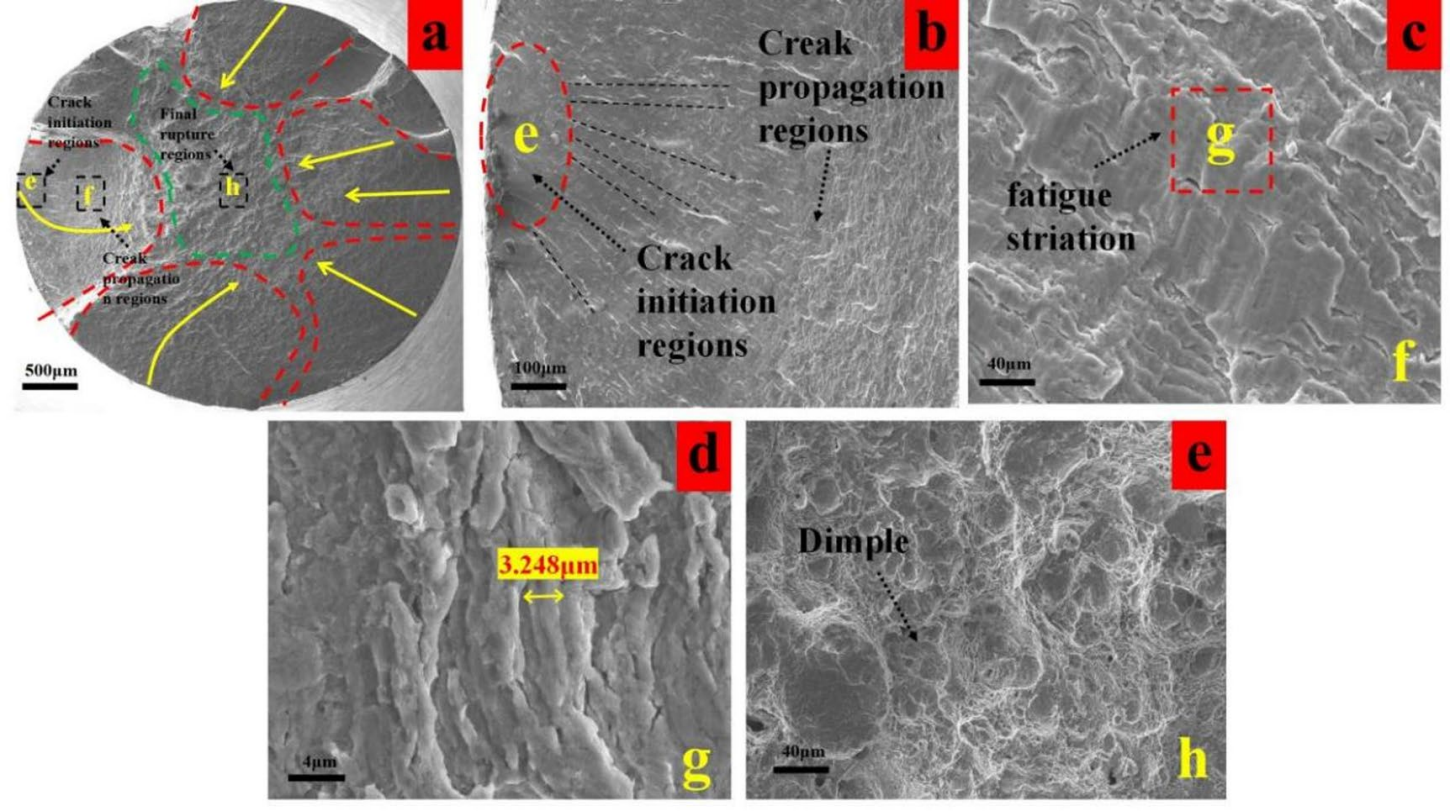
Fatigue fracture analysis of group T4 (**a**) macro-morphology of fracture, (**b**) enlarged view of crack initiation region, (**c**) fatigue striation of crack propagation zone, (f) dimples in the final rupture region.

As shown in Fig. 14a, the fracture surface of the T4 specimen macroscopically exhibits a discernible crack propagation zone. Although the proportion of the fatigue area is close to that of the T1 specimen, the high surface roughness (0.255 μm) of T4 activated multiple micro-defects simultaneously, forming at least 4–5 fatigue initiation sites. These machining-induced defects created deep, tear-like pits on the surface, significantly shortening the fatigue propagation zone. Furthermore, the shallow effective compressive residual stress gradient layer accelerated the completion of the crack initiation stage.

As illustrated in Fig. 14b, the crack propagation marks are widely spaced and discontinuous, while the final fracture zone dominates the fracture surface. This indicates that the transition from crack initiation to rapid propagation occurred extremely quickly, with virtually no effective inhibition, corresponding directly to the short fatigue life (Fig. 11). In the propagation zone, the fatigue striations are coarse, unevenly distributed, and locally discontinuous (Fig. 14c,d), suggesting a very high crack propagation rate.

The final fracture area exhibits coarse and non-uniformly distributed dimples (Fig. 14e). This morphology results from the rapid crack growth, which caused accelerated internal damage accumulation and limited plastic deformation during final fracture, leading to a state of “inadequate ductility” in the fracture characteristics.

**Fig. 15:**
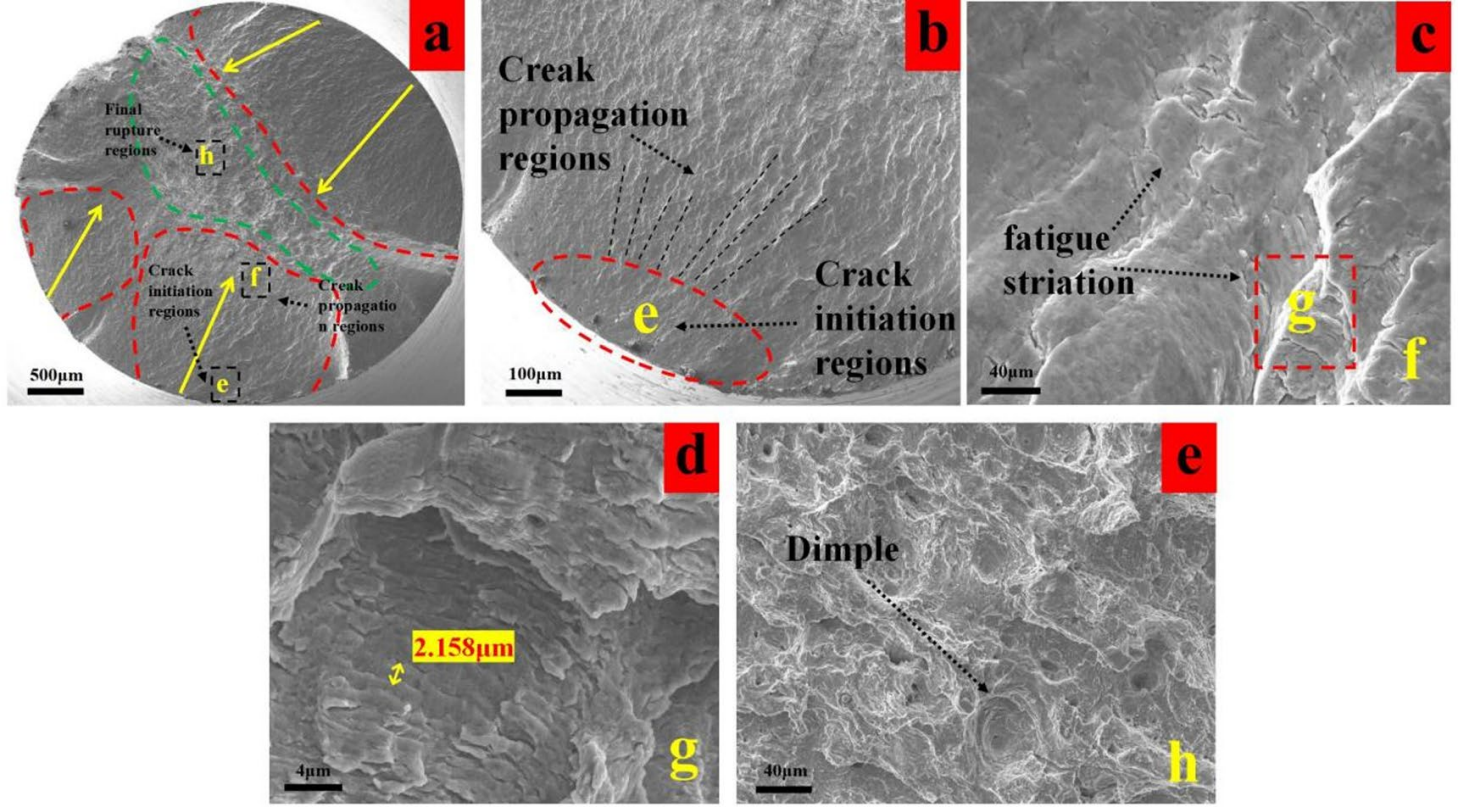
Fatigue fracture analysis of group T5 (**a**) macro-morphology of fracture, (**b**) enlarged view of crack initiation region, (**c**) fatigue striation of crack propagation zone, (f) dimples in the final rupture region.

As shown in Fig. 15a,b, the fatigue life of the T5 specimen lies between those of T1 and T4. Although it possesses the optimal surface roughness (0.111 μm), its shallow residual stress gradient results in fracture characteristics indicative of a balanced state: weak suppression of crack initiation but relatively stable crack propagation. Clear crack propagation marks are observed. Despite the low surface roughness, the shallow stress gradient provides insufficient inhibition of crack initiation, leading to a slightly larger initiation zone.

The fatigue striations in the propagation zone (Fig. 15c,d) exhibit a density intermediate between those of T1 and T4, with good striation continuity. This suggests that the moderate compressive residual stress (−506.7 MPa) effectively and steadily hindered crack propagation, compensating for the deficiency in the initiation stage and resulting in intermediate overall fatigue life. The dimples in the final fracture zone are uniformly sized (Fig. 15e), falling between those of T1 and T4, indicating reasonably preserved material plasticity without significant damage accumulation due to the shallow stress gradient. This observation is consistent with the moderate degree of surface layer grain refinement (Fig. 10c).

**Fig. 16:**
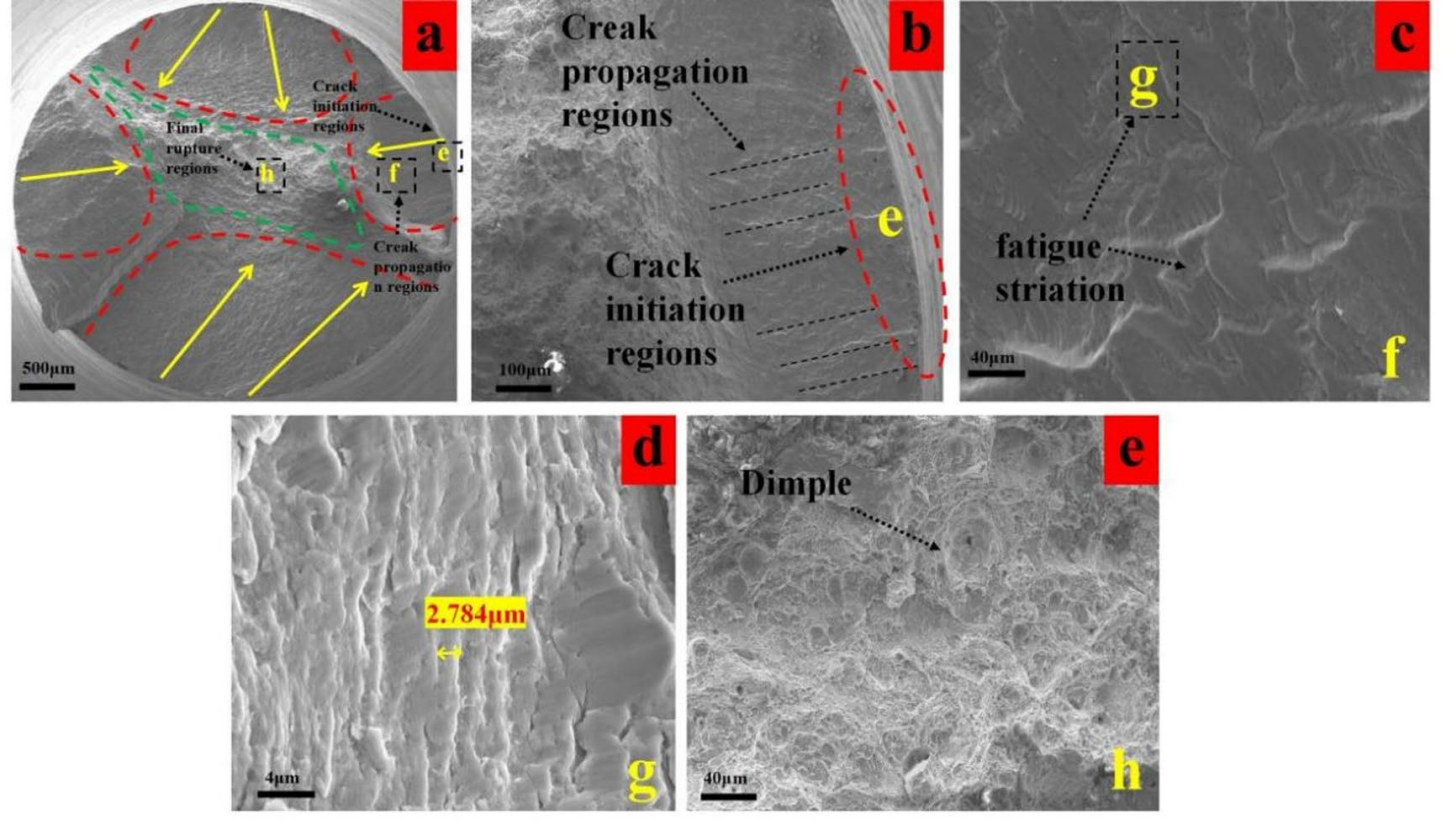
Fatigue fracture analysis of group T8 (**a**) macro-morphology of fracture, (**b**) enlarged view of crack initiation region, (**c**) fatigue striation of crack propagation zone, (f) dimples in the final rupture region.

As shown in Fig. 16a,b, although the T8 specimen exhibits higher surface roughness (0.391 μm), its deeper residual stress gradient effectively suppresses crack propagation, thereby compensating for the adverse effect of poor surface roughness during the initiation stage. This results in a fatigue life comparable to that of the T5 specimen. The deep stress gradient inhibits the propagation of secondary cracks, offsetting the disadvantage caused by high surface roughness.

Fatigue striations in the crack propagation zone display spacing similar to those in T5, with slightly better striation integrity observed in T8 (Fig. 16c,d). This indicates that the deep stress gradient provides more sustained resistance to crack advancement, effectively counteracting the accelerated propagation tendency induced by surface defects.

The final fracture zone exhibits slightly non-uniform dimple sizes (Fig. 16e), suggesting that micro-defects associated with high surface roughness only marginally affect plastic deformation distribution during the final fracture stage. Nevertheless, the preservation of overall material toughness by the deep stress gradient ensures that the fracture remains predominantly ductile in nature.

The comparison between T5 and T8 provides compelling evidence that a favorable residual stress distribution can effectively compensate for the detrimental effects of poor surface roughness. Conversely, superior surface roughness alone fails to fully realize its potential advantage in the absence of adequate residual stress support. This finding directly validates the rationality of assigning a higher weighting factor to residual stress in the weighted standard value.

### Basic theoretical analysis

**Fig. 17:**
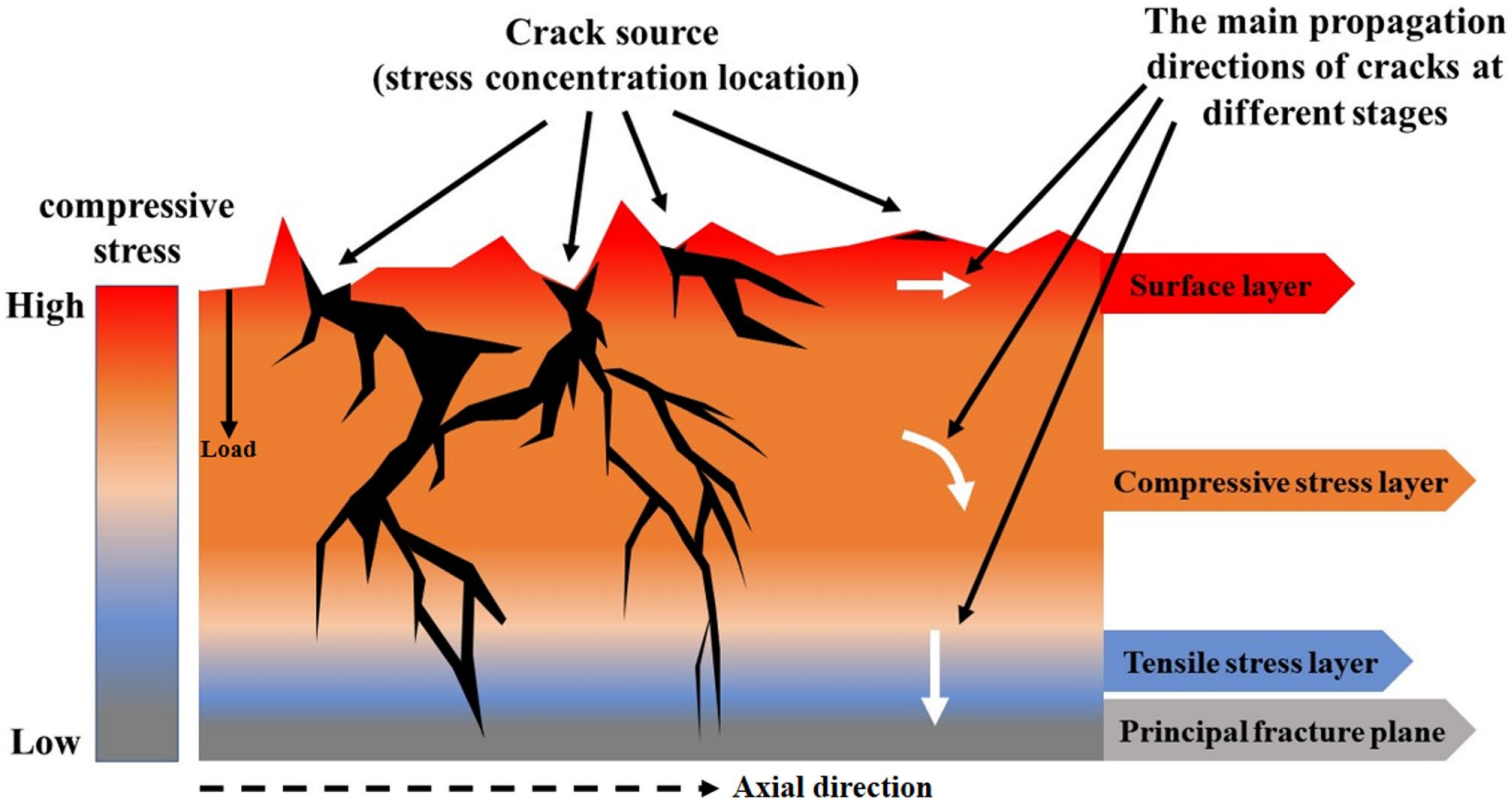
Analysis of the effects of roughness and residual stress on crack propagation.

As shown in Fig. 17, the influence of surface roughness and residual compressive stress on cracks can be categorized into three primary aspects: the initiation of surface cracks, the suppression of near-surface cracks, and the inhibition of deep crack propagation.

The initiation of surface cracks is predominantly governed by surface roughness, which determines the number of crack origins. Higher surface roughness facilitates the formation of multiple fatigue initiation sites under cyclic loading, with these sites typically located at stress concentration points. The suppression of surface and sub-surface cracks is primarily dominated by surface residual compressive stress. This stress effectively inhibits crack initiation. Even at locations with significant stress concentration, the presence of high-magnitude surface residual compressive stress can cause the crack to propagate along the axial direction or in a direction nearly parallel to it, thereby significantly reducing the propagation rate. In some cases, it may even lead to the closure of micro-cracks, arresting their propagation. The inhibition of deep crack propagation is mainly governed by the compressive stress gradient layer. In deeper regions, cracks initially propagate along the axial direction or in a direction nearly parallel to it. However, as the depth increases and the compressive stress layer gradually diminishes, cracks tend to transition to propagation along the loading direction. Eventually, fracture may occur in the core tensile stress layer.

In summary, the fatigue fracture of the specimens is jointly influenced by surface roughness and residual stress. However, the dominant role of residual stress outweighs that of surface roughness. Even under poor surface roughness conditions (e.g., Group T8), sufficient surface residual compressive stress and its favorable gradient distribution can compensate for the adverse effects of roughness by inhibiting crack propagation, thereby maintaining a high fatigue life.

## Conclusion

This study systematically investigated the influence of cutting parameters—cutting speed (v), feed rate (f), and depth of cut ($\:{\mathrm{a}}_{\mathrm{p}}$)—on the surface integrity and fatigue performance of quenched and tempered 42CrMo steel. Through single-factor experiments and rotating bending fatigue tests, the feasibility of using the weighted standard value to represent fatigue life was validated. Furthermore, a comprehensive evaluation of the effects of cutting parameters and surface integrity on fatigue life was established, as summarized in Table 5.

**Table 5:** The impact of cutting parameters and surface integrity on evaluating fatigue life.

Cutting parameters		Surface integrity		Fatigue life evaluation	
		Roughness(μm)	Residual stress(MPa)	Fatigue life	Weighted standard value
		R_a_	*σ*	*N*	X_i_
Feed rate(mm/rev)	*f*	◎	●	●	●
Trend (↑)		(↑)	(↓)	(-)	(↓)
Depth of cut(mm)	a_p_	○	○	○	○
Trend (↑)		(↑)	(↑↓)	(-)	(↓)
Cutting speed(m/min)	*v*	●	◎	●	●
Trend (↑)		(↓)	(↓)	(-)	(↑)

●indicates significant influence; ◎ indicates moderate influence; ○ indicates minor or negligible influence; ↑ indicating an increasing trend; ↓ indicating a decreasing trend; ↑↓ indicating a trend of first increasing and then decreasing; - indicating insufficient data.

The main conclusions drawn from this study are as follows:

1. The cutting speed exerts the most significant influence on surface roughness. Machining within the low to medium cutting speed range notably improves surface quality, while even at higher speeds combined with cutting fluid, good surface finish can be maintained. The feed rate has a moderate effect on surface roughness, as its increase leads to a greater theoretical residual height, thereby elevating the roughness value. In contrast, the depth of cut shows a minor influence on roughness, with slow variation trends and negligible fluctuations.
2. The residual stress distribution exhibits the most pronounced dependence on the cutting speed. High-speed cutting combined with effective cooling from the cutting fluid promotes a synergistic interaction dominated by intense mechanical plastic deformation with suppressed thermal effects. This mechanism results in high-magnitude residual compressive stresses and a deeper stress gradient layer, which significantly inhibit the initiation and propagation of fatigue cracks.
3. Microstructural alterations exhibit a strong correlation with the cutting speed. Higher cutting speeds induce more pronounced grain refinement and a deeper plastically deformed layer. This effect is most prominent under the T1 parameter set, where the surface layer undergoes the most significant microstructural refinement, thereby further enhancing the material’s fatigue resistance.
4. The initiation and propagation of cracks are governed by the synergistic effects of surface roughness and residual stress. Surface residual compressive stress and its gradient distribution serve as the dominant factors influencing fatigue life. The specimen with optimal parameter T1 achieved the highest fatigue life of 95,220 cycles, validating that high residual compressive stress combined with a deep gradient layer plays a critical role in enhancing fatigue performance.
5. The weighted standard value proposed in this study, which integrates surface roughness and residual compressive stress, demonstrates strong consistency with the results of rotating bending fatigue tests. This confirms its effectiveness as a reliable indicator for evaluating the fatigue life of quenched and tempered 42CrMo steel.

This study provides experimental evidence and theoretical support for optimizing the cutting parameters of quenched and tempered 42CrMo steel, thereby enhancing the fatigue life of critical components. The comprehensive evaluation method offers a practical and reliable theoretical tool for improving component service performance through the optimization of cutting parameters.

## Data Availability

The datasets generated and/or analysed during the current study are not publicly available due [ue to confidentiality agreements in our lab, we are unable to share the raw data at this time.] but are available from the corresponding author on reasonable request.
